# Evaluating the Performance of qVFM in Mapping the Visual Field of Simulated Observers With Eye Diseases

**DOI:** 10.3389/fnins.2021.596616

**Published:** 2021-06-21

**Authors:** Pengjing Xu, Luis Andres Lesmes, Deyue Yu, Zhong-Lin Lu

**Affiliations:** ^1^College of Optometry, The Ohio State University, Columbus, OH, United States; ^2^Shanghai Technology Development Co., Ltd., Shanghai, China; ^3^Shanghai-Warsaw Joint Laboratory on Artificial Intelligence, Shanghai, China; ^4^Adaptive Sensory Technology, Inc., San Diego, CA, United States; ^5^Division of Arts and Sciences, NYU Shanghai, Shanghai, China; ^6^Center for Neural Science, Department of Psychology, New York University, New York, NY, United States; ^7^NYU-ECNU Institute of Cognitive Neuroscience at NYU Shanghai, Shanghai, China

**Keywords:** Bayesian adaptive testing, active learning, perimetry, visual-field map, scotoma, glaucoma, age-related macular degeneration, cataract

## Abstract

**Purpose:**

Recently, we developed a novel active learning framework, qVFM, to map visual functions in the visual field. The method has been implemented and validated in measuring light sensitivity and contrast sensitivity visual field maps (VFMs) of normal observers. In this study, we evaluated the performance of the qVFM method in mapping the light sensitivity VFM of simulated patients with peripheral scotoma, glaucoma, age-related macular degeneration (AMD), and cataract.

**Methods:**

For each simulated patient, we sampled 100 locations (60 × 60 degrees) of the visual field and compared the performance of the qVFM method with a procedure that tests each location independently (the qYN method) in a cued Yes/No task. Two different switch modules, the distribution sampling method (DSM) and parameter delivering method (PDM), were implemented in the qVFM method. Simulated runs of 1,200 trials were used to compare the accuracy and precision of the qVFM-DSM, qVFM-PDM and qYN methods.

**Results:**

The qVFM method with both switch modules can provide accurate, precise, and efficient assessments of the light sensitivity VFM for the simulated patients, with the qVFM-PDM method better at detecting VFM deficits in the simulated glaucoma.

**Conclusions:**

The qVFM method can be used to characterize residual vision of simulated ophthalmic patients. The study sets the stage for further investigation with real patients and potential translation of the method into clinical practice.

## Introduction

Standard automated perimetry (SAP) (Goldmann, 1945a,b; Harms, 1952; Aulhorn and Harms, 1972; Lachenmayr et al., 1994; Rogers and Landers, 2005; Milner and Goodale, 2006; Strasburger et al., 2011) is used to assess the light sensitivity visual field map (VFM) in routine clinical eye exams to detect and manage a number of eye diseases that cause visual field deficits, including glaucoma (Caprioli, 1991; Smith et al., 1996; Ng et al., 2012), peripheral scotoma caused by a number of pathogenesis (Portney and Krohn, 1978; Fendrich et al., 1992), age-related macular degeneration (AMD) (Anderson et al., 2011; Luu et al., 2013), cataract (Radius, 1978; Lam et al., 1991), retinitis pigmentosa (Jacobson et al., 1986; Iannaccone et al., 1995), cytomegalovirus retinitis (Bachman et al., 1992; Thorne et al., 2011), stroke (Townend et al., 2007), and other neurological deficits (Papageorgiou et al., 2007). However, the assessment of VFM based on SAP is very noisy (Heijl et al., 1989). To improve the precision of light sensitivity VFM in automated perimetry and enable assessments of other visual functions, we recently developed a novel active learning framework, the qVFM method, that combines a global module for preliminary assessment of the shape of the VFM and a local module for assessing visual function at individual visual field locations (Xu et al., 2018, 2019a,b, 2020). Both computer simulations and psychophysical validation studies that tested the light sensitivity VFM of 12 eyes of six normal observers (Xu et al., 2019a) and contrast sensitivity VFM of 10 eyes of five normal observers (Xu et al., 2020) showed that the qVFM method could provide accurate, precise and efficient VFM assessments.

In Xu et al. (2019a), we compared the qVFM method with the conventional staircase-based SAP methods. We showed that the conventional staircase-based SAP methods exhibited considerable larger biases and variabilities than the qVFM. The focus of the current study is to evaluate the potential of the qVFM method in mapping the light sensitivity VFM of simulated patients with peripheral scotoma, glaucoma, AMD, and cataract. Specifically, we simulated a scotoma observer with three scotomas located in the periphery, a glaucoma observer with peripheral vision deficits outside of the fovea, an AMD observer with deficits at the fovea, and a cataract observer with lower light sensitivity across the entire visual field. This is the first step in our attempt toward evaluating the qVFM method in clinical populations. We plan to further evaluate and translate the qVFM method into clinical practice in the future.

The qVFM method consists of three modules, a preliminary assessment of the general shape of the VFM (the global module), an assessment of visual functions at each individual visual field location (the local module), and a switch module that determines when to switch from the global module to the local module. The global module is used to estimate the overall shape of the visual field in the beginning of the assessment, and the local module is used to provide a detailed location-by-location characterization of the VFM based on priors generated from the global module. Given that the goal of clinical VFM assessment is to detect deviations from the normal VFM, it is essential to assess the performance of the qVFM method in measuring pathological visual fields with characteristic patterns that deviate severely from the normal VFM. Our hypothesis is that even though the global module does not provide a complete model of the detailed structure of the VFM in some severe cases, it still provides a reasonable approximation, and then the local module can swiftly take over to measure the detailed local structure of the VFM. We also compared two different switching methods in this study, one based on a distribution sampling method (DSM) (Xu et al., 2018, 2019a), and the other a newly developed procedure based on a parameter delivering method (PDM).

## Methods

### qVFM Implementation

Developed in Xu et al. (2019a,b, 2020), the qVFM method consists of three major modules (see Supplementary Appendix B for more details):

(1) The global module, which measures the shape of the VFM modeled as a tilted elliptic paraboloid function (TEPF) with five parameters (Eq. 1). The score at each visual field location represents a measure of visual function (e.g., light sensitivity, contrast sensitivity) at that location.
(2) The switch module, which evaluates the rate of information gain in the global module and determines when to switch to the local module. At the switching point, the module generates a prior distribution of the measure of visual function at each visual field location based on the posterior distribution from the global module.
(3) The local module, which uses the prior generated by the switch module to provide assessment of visual function at each visual field location. It uses another Bayesian adaptive procedure that determines the order and test stimulus based on the relative information gain across locations.

The global module models and assesses the global shape of light sensitivity VFM as a TEPF:

(1) $$\tau \,\left(x,y\right)=E\,P\,Z-{\left(\frac{x}{E\,P\,A}\right)}^{2}-{\left(\frac{y}{E\,P\,B}\right)}^{2}+S\,L\,A*x+S\,L\,B*y,$$

where *EPZ* (unit: dB) is the light sensitivity at the fovea, *EPA* (unit: degree/$\sqrt{d\,B}$) is the root bandwidth in the horizontal direction of the light sensitivity VFM, *EPB* (unit: degree/$\sqrt{d\,B}$) is the root bandwidth in the vertical direction, *SLA* (unit: dB/degree) is the horizontal tilt level of the light sensitivity VFM, and *SLB* (unit: dB/degree) is the vertical tilt level. The height of the TEPF, τ(*x*,*y*), is the light sensitivity (unit: dB) at visual field location *(x, y)* at *d*′ = 1.0.

A Yes/No (YN) task was adapted in this study, which means that the probability of reporting target presence is determined by both the light sensitivity and decision criterion. After introducing the sixth parameter λ for decision criterion, the global model can predict the overall probability of light detection across the visual field $p\,\left(\vec{\theta }\right)$, where $\vec{\theta }=\left(E\,P\,Z,E\,P\,A,E\,P\,B,S\,L\,A,S\,L\,B,\lambda \right)$, with a fixed slope of the psychometric function. A prior distribution *p*_*t* =__ 0_($\vec{\theta }$) is defined based on *a priori* knowledge of the VFM before any data collection. In addition, all possible stimulus intensities and stimulus locations *(x, y)* are included in the stimulus space. The optimal stimulus in the next trial, which would generate the maximum expected information gain, is determined *via* a one-step-ahead search strategy. After receiving the response from the observer, the posterior distribution of the parameters is updated using Bayes rule (Kontsevich and Tyler, 1999; Lesmes et al., 2006, 2010, 2015).

Since the global module cannot estimate the detailed structure of the VFM, a local module is necessary for a more detailed assessment. The switch module determines the switching point and also sets the prior distributions for the local module. Two different switch methods were implemented in this study, the DSM (Xu et al., 2018, 2019a) and the newly developed PDM. In this study, the prior distribution in the local module was defined with a two-dimensional probability distribution of light sensitivity and decision criterion at each visual field location. The DSM and PDM used the same trend of expected information gain in the global model to determine the switching point, but different procedures to generate the prior distributions for the local module from the six-dimensional posterior distribution in the global module.

The DSM samples the posterior distribution in the global module repeatedly to generate the prior distributions at each visual location in the local module, with 1,600 samples per location. The PDM computes the means of the marginal posterior distributions of the five parameters (EPZ, EPA, EPB, SLA, and SLB) of the TEPF model, and sets the expected value of the prior for light sensitivity, τ(x,y), at each visual field location based on the TEPF model. It also sets the expected value of the prior of decision criterion λ at each visual field location, using its mean of the marginal posterior distribution from the global module. It then uses the average 68.2% half width of the credible interval (HWCI) of the posterior distributions of the estimated light sensitivities and decision criterions across all visual field locations to set the variability of the prior distributions in the local module. Specifically, a hyperbolic secant (sech) function (King-Smith and Rose, 1997) is used to set up the prior distributions. For each parameter θ*_*i*_* (i = 1, 2), the mode of the marginal prior *p*(θ*_*i*_*) is defined by the expected value of the corresponding parameter, θ*_*i,guess*_*, from the posterior distributions of the global module, and the width is defined by the 68.2% credible interval of that parameter.

(2) $$P\left(\theta_{i}\right)=\text{sech[}\theta_{i,c\,o\,n\,f\,i\,d\,e\,n\,c\,e}\times \left(\theta_{i}-\theta_{i,g\,u\,e\,s\,s}\right)],$$

where:

(3) $$\text{sech}\,\left(\text{z}\right)=\frac{2}{e^{z}+e^{-z}}.$$

The joint prior is defined as the normalized product of the marginal priors of light sensitivity and decision criterion, generated for each visual field location in the local module.

The qYN procedure (Lesmes et al., 2015) is used to estimate the posterior distribution of the two parameters at each visual field location in the local module. It is also used as a reduced qVFM procedure that has only the local module for performance comparison with the full qVFM procedure that has all three modules.

### Simulating Observers With Eye Diseases

In this study, we simulated the VFM of the OS eye of five observers: one normally sighted, and four with peripheral scotoma, glaucoma, AMD, or cataract.

The parameters of the normal observer were the same as those used in Xu et al. (2019a). Table 1 lists the values of EPA, EPB, EPZ, SLA, SLB, λ and the average SDs of the corresponding parameters from the 12 eyes of six normal observers tested in that study.

**TABLE 1:** Parameters of the normal observer: EPA (unit: degree/$\sqrt{d\,B}$), EPB (unit: degree/$\sqrt{d\,B}$), EPZ (unit: dB), SLA (unit: dB/degree), SLB (unit: dB/degree), and λ.

Parameters	EPA	EPB	EPZ	SLA	SLB	λ
Value	81.0	41.1	24.3	0.020	0.032	1.20
SD	2.4	2.9	0.62	0.035	0.045	0.14

*In this study, the dB values were calculated as *−*10 × log10[luminance (in asb)/10000].*

The blind spot of the simulated OS eye was at 15 degrees left and three degrees below the fovea, i.e., (*−*15, 3). At the stated coordinate, each point represented a 6-degree square region. For the four simulated observers with eye diseases, the parameters were modified from those of the normal observer: (1) The simulated scotoma observer had three scotomas, located at (9, 9), (*−*9, 9), and (*−*9, 15). (2) The simulated glaucoma observer had defective peripheral vision outside of the central 12 × 15 degrees rectangle area from the upper-left (*−*6, *−*6) to the lower-right (6, 9), in which light sensitivity was 2.5 times lower than that of the normal observer. (3) The simulated AMD observer had poor foveal vision in the central 12 × 15 degrees rectangle area from the upper-left (*−*6, *−*6) to the lower-right (6, 9), in which light sensitivity was 12.3 dB, about 12 dB lower than that of the normal observer. (4) The simulated cataract observer had 1.7 times lower light sensitivity than the normal observer across the entire visual field.

In the simulations, observers performed the light detection task described in Xu et al. (2019a). Briefly, the test target was a small light disc with a 0.43-degree diameter with luminance between 31.5 and 950 asb (corresponding to 10.2–25.0 dB). Each trial began with a potential 150-ms target at one of the 100 cued visual field locations. Simulated observers were asked to indicate the presence or absence of the target, with the luminance of the target determined by an adaptive procedure in each trial. Their response in each trial was determined by their light sensitivity VFM defined by the simulation procedure, which was unknown to the qVFM procedure that was used to estimate their light sensitivity VFM. The performance of the full qVFM procedure, with both the DSM and PDM switch modules, was compared with that of the qYN procedure, which assessed light sensitivity at each location independently, in 200 repeated simulations of 1,200 trials each.

### Evaluation Metrics

We quantified the accuracy of the estimated VFMs using the root mean squared error (RMSE) of the estimated sensitivities across all 100 visual field locations. RMSE after the i-th trial can be calculated as:

(4) $${\text{RMSE}}_{i}=\sqrt{\frac{\sum_{k}\sum_{j}{\left(\tau_{i\,j\,k}-\tau_{k}^{t\,r\,u\,e}\right)}^{2}}{J\times K}},$$

where τ_*ijk*_ is the estimated sensitivity at the k-th visual field location after the i-th trial in the j-th run, and τ_*k*_^*true*^ is the true sensitivity at that location.

Two methods were used to assess the precision of the qVFM procedure. The first is based on the standard deviation (SD) of repeated measures:

(5) $${\text{SD}}_{i}=\sqrt{\frac{\sum_{k}\sum_{j}{\left[\tau_{i\,j\,k}-m\,e\,a\,n\,\left(\tau_{i\,j\,k}\right)\right]}^{2}}{J\times K}}.$$

The second is the HWCI of the posterior distributions of the estimated sensitivities. The 68.2% credible interval represents the range within which the actual value lies with 68.2% probability, representing an interval that contains the true value of the parameter in 68.2% of unlimited repetitions.

Global indices on the estimated VFMs were also adapted and calculated for each method, including mean defect, loss variance, short-term fluctuation and corrected loss variance (Flammer et al., 1985). These metrics are used in the clinic to quantify diffuse depression, local defects, and scatter observed during VFM tests as well as local inhomogeneity of visual field defects.

The mean defect (MD) of the estimated sensitivities across all 100 visual field locations after the i-th trial is calculated as:

(6) $${\text{MD}}_{i}=\frac{\sum_{k}\sum_{j}\left(\tau_{k}^{t\,r\,u\,e}-\tau_{\text{ijk}}\right)}{J\times K},$$

The loss variance (LV) is calculated as:

(7) $${\text{LV}}_{i}=\frac{\sum_{k}\sum_{j}{\left(\tau_{\text{ijk}}-\tau_{k}^{t\,r\,u\,e}+{\text{MD}}_{i}\right)}^{2}}{J\times \left(K-1\right)}.$$

The short-term fluctuation (SF) is calculated as:

(8) $${\text{SF}}_{i}=\sqrt{\frac{\sum_{k}\sum_{j}{\left(\tau_{i\,j\,k}-m\,e\,a\,n\,\left(\tau_{i\,j\,k}\right)\right)}^{2}}{\left(J-1\right)\times K}}.$$

The corrected loss variance (CLV) is calculated as:

(9) C⁢L⁢Vi=LVi-SFi2.

## Results

**FIGURE 1:**
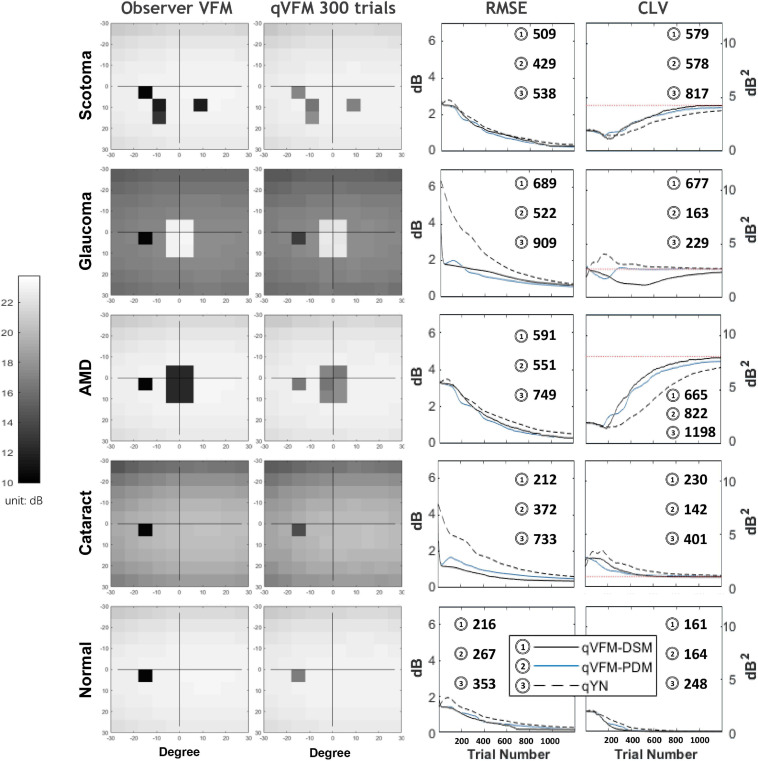
Summary results from the five simulated observers across 200 runs. The true VFM of each simulated observer (monocular) is presented in the first column with an achromatic colormap. The estimated VFMs obtained with the qVFM-PDM method after 300 trials are presented in the second column. The corresponding root mean squared error (RMSE) and corrected loss variance (CLV) of the estimates as functions of trial number are shown in the third and fourth columns. The trial numbers needed to achieve 1 dB RMSE and 1 dB^2^ within CLV are shown in the corresponding subplots for the three methods.

We present the simulation results for the scotoma, glaucoma, AMD, cataract, and normal observers in the following sections. Figure 1 provides a summary of the major results.

### Simulated Scotoma Observer

**FIGURE 2:**
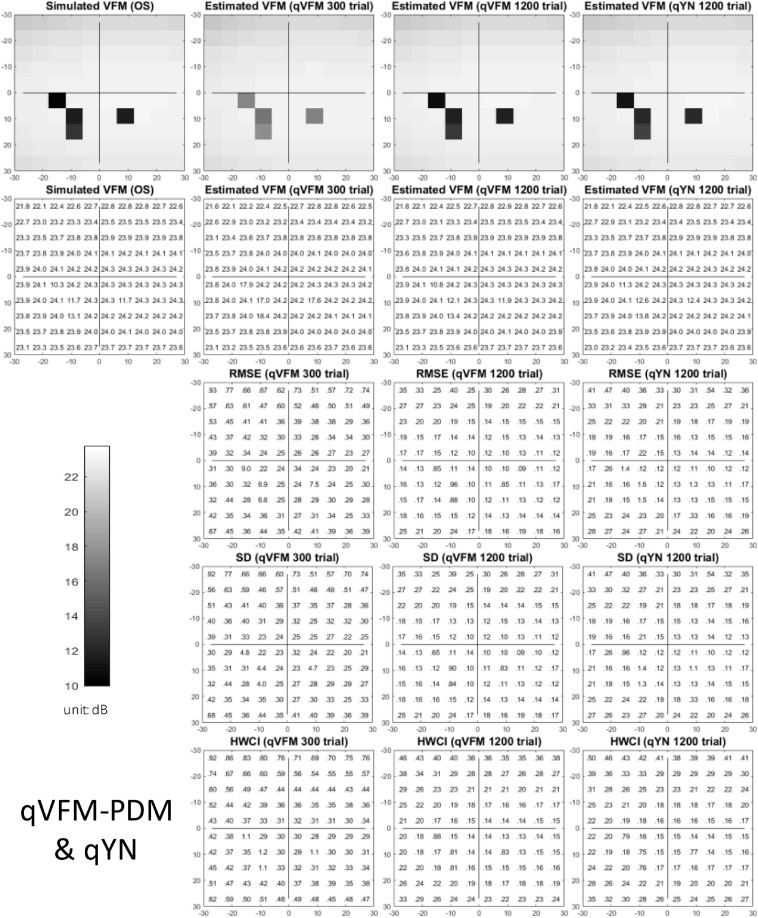
Simulation results I of the scotoma observer across 200 runs. The true VFM of the simulated observer (monocular) is presented in the first column of the first row with achromatic colormaps and second row with numerical values. The estimated VFMs obtained with the qVFM-PDM method after 300 trials and 1,200 trials, qYN method after 1,200 trials are presented in the first and second rows, respectively. The corresponding RMSE, SD, and 68.2% HWCI of the estimates are in the third, fourth, and fifth rows.

The estimated light sensitivity VFMs, the corresponding RMSE, standard deviation and average 68.2% HWCI for the simulated scotoma observer, obtained from the qVFM-PDM methods are shown in Figure 2, along with results from the qYN method. The corresponding results from the qVFM-DSM are shown in Supplementary Figure A1.

Compared with the simulated normal observer, the average light sensitivity deficit across the three scotoma locations is 12.0 dB for the simulated scotoma observer. Across the three scotoma locations, the average estimated deficits are 7.79 ± 5.70 dB and 11.9 ± 1.21 dB after 300 and 1,200 trials with the qVFM-DSM method, 6.53 ± 4.43 dB and 11.8 ± 0.86 dB after 300 and 1,200 trials with the qVFM-PDM method, and 11.3 ± 1.34 dB after 1,200 trials with the qYN method.

**FIGURE 3:**
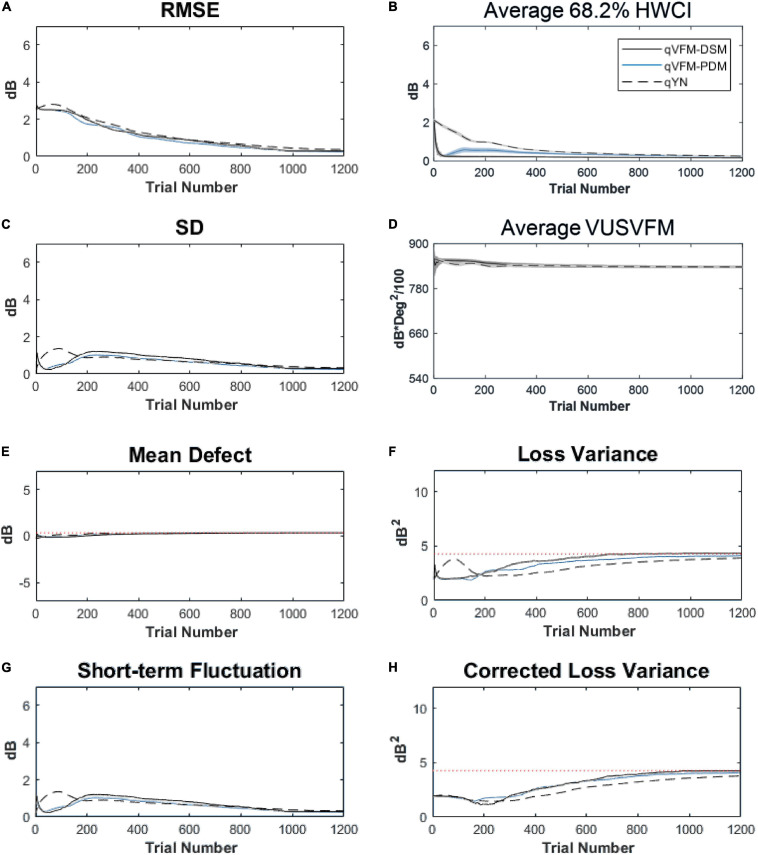
Performance I of the qVFM-DSM, qVFM-PDM and qYN methods in estimating VFM of the simulated scotoma observer across 200 runs. **(A)** Average root mean squared error, **(B)** Average 68.2% HWCI of the estimated VFM, **(C)** Average standard deviation, **(D)** Average volume under the surface of the VFM (VUSVFM), **(E)** Mean defect, **(F)** Short-term fluctuation, **(G)** Loss variance, and **(H)** Corrected loss variance. Results from the qVFM-DSM and qVFM-PDM methods are shown in solid black and blue lines, and results from the qYN method are shown in dashed lines. The true values of the global indices are shown in red dotted lines. For panels **(B,D)**, shaded regions represent ± 1 SD of the corresponding value.

For the trial-by-trial performance, the RMSE, the average 68.2% HWCI, SD, the mean defect, the loss variance, the short-term fluctuation and the corrected loss variance of the estimated light sensitivity VFM from the qVFM-DSM, qVFM-PDM, and qYN methods are shown in Figures 3A–C,E–H, with numerical results listed in Table 2.

**TABLE 2:** RMSE, SD, average 68.2% HWCI, mean defect, loss variance and corrected loss variance of the estimated VFM from the qVFM-DSM, qVFM-PDM and qYN methods in the beginning (0 trial), after 300 and 1,200 trials are listed for the simulated scotoma observer.

Scotoma	Trial number	0	300	1,200	True value	1 dB/dB^2^ threshold
RMSE (dB)	qVFM-DSM	2.54	1.42	0.29		509 ± 70
	qVFM-PDM	2.54	1.57	0.25		429 ± 40
	qYN	2.54	1.71	0.37		538 ± 33
SD (dB)	qVFM-DSM	0	1. 15	0. 29		
	qVFM-PDM	0	0.99	0. 24		
	qYN	0	0.89	0. 33		
HWCI (dB)	qVFM-DSM	2.12	0. 22	0.17		
	qVFM-PDM	2.12	0. 48	0.24		
	qYN	2.12	0. 73	0.26		
Mean defect (dB)	qVFM-DSM	*−*0.26	0.20	0.36	0.36	
	qVFM-PDM	*−*0.26	0.19	0.36	0.36	
	qYN	*−*0.26	0.26	0.36	0.36	
Loss variance (dB^2^)	qVFM-DSM	1.95	3.27	4.33	4.28	
	qVFM-PDM	1.95	2.84	4.15	4.28	
	qYN	1.95	2.31	3.89	4.28	
Corrected loss variance (dB^2^)	qVFM-DSM	1.95	1.94	4.24	4.28	579 ± 68
	qVFM-PDM	1.95	1.86	4.15	4.28	578 ± 61
	qYN	1.95	1.50	3.79	4.28	817 ± 69

*The corresponding true values are listed in the sixth column. Trial numbers needed to achieve 1 dB RMSE and 1 dB^2^ within the corrected loss variance are listed with their SD in the last column.*

In characterizing spatial vision, the area under the log contrast sensitivity function is often used as a summary metric (Applegate et al., 1998, 2000; Oshika et al., 1999, 2006; van Gaalen et al., 2009). In Figure 3D, we show the average volume under the surface of the VFM (VUSVFM) across 200 iterations of the simulation to provide a summary metric of the entire visual field for the simulated scotoma observer.

For the simulated scotoma observer, the results show that the qVFM-DSM and qVFM-PDM methods have similar performance. In addition, both the qVFM-DSM and qVFM-PDM methods demonstrate better efficiency than the qYN method. The trial numbers needed to achieve 1 dB RMSE and 1 dB^2^ within the corrected loss variance are shown in the last column of Table 2.

**FIGURE 4:**
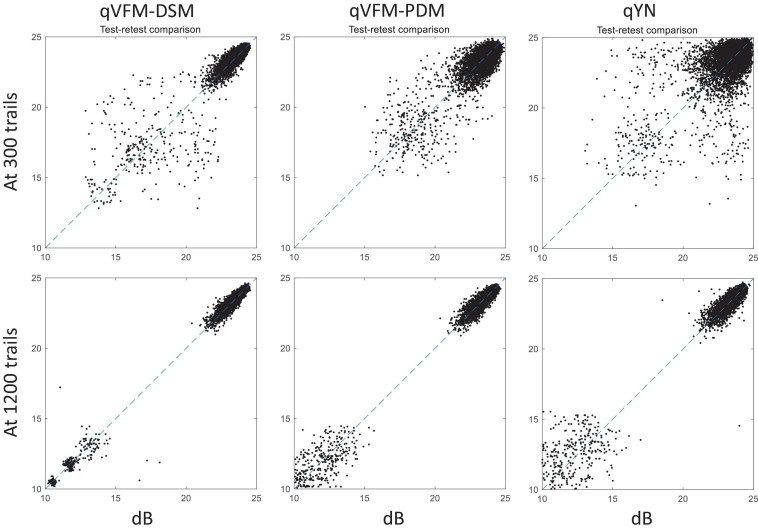
Performance II of the qVFM-DSM, qVFM-PDM, and qYN methods in estimating VFM of the simulated scotoma observer. Test-retest comparison of the estimated light sensitivities from repeated 200 runs at 300 and 1,200 trials.

For the simulated scotoma observer, test–retest reliabilities of the three methods are assessed through analysis of VFM estimates at 300 and 1,200 trials across 200 runs (Figure 4). Each subplot displays estimated sensitivities (at 100 VFM locations) of the 100 paired runs (100 locations × 100 random pairs of runs = 10,000 data points). The average test–retest correlations for the paired VFM estimates at 300 trials are 0.909 (SD = 0.002) for the qVFM-DSM, 0.842 (SD = 0.003) for the qVFM-PDM and 0.592 (SD = 0.008) for the qYN methods, respectively. The average correlations at 1,200 trials are 0.994 (SD = 0.0001) for the qVFM-DSM, 0.99 (SD = 0.0003) for the qVFM-PDM and 0.98 (SD = 0.0005) for the qYN methods, respectively.

### Simulated Glaucoma Observer

**FIGURE 5:**
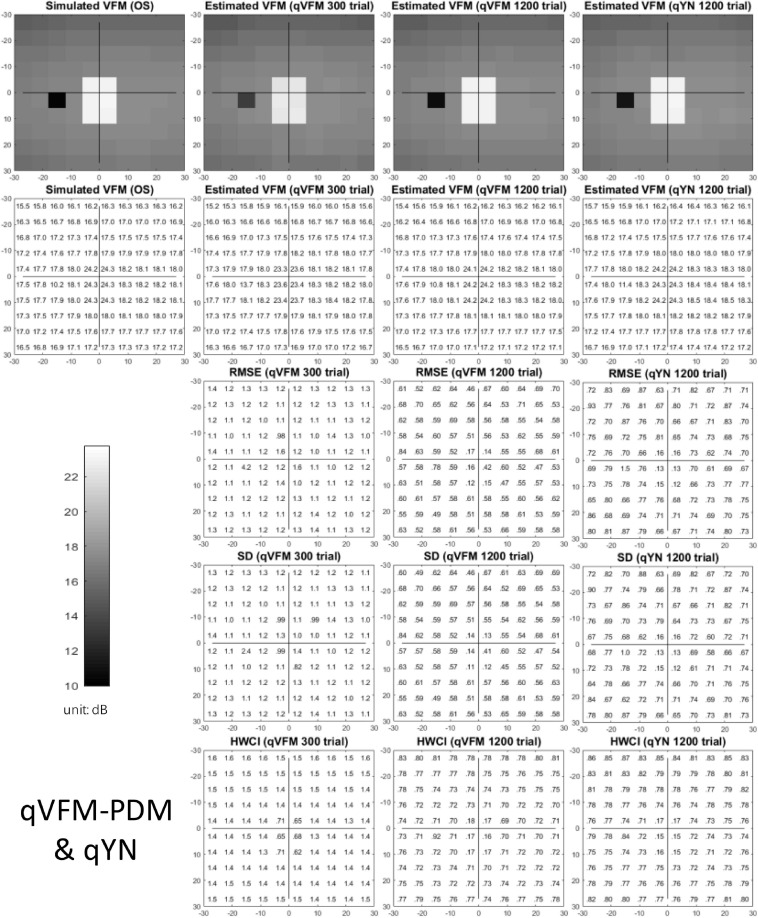
Simulation results I of the glaucoma observer across 200 runs. The true VFM of the simulated observer (monocular) is presented in the first column of the first row with achromatic colormaps and second row with numerical values. The estimated VFMs obtained with the qVFM-PDM method after 300 trials and 1,200 trials, qYN method after 1,200 trials are presented in the first and second rows, respectively. The corresponding RMSE, SD, and 68.2% HWCI of the estimates are shown in the third, fourth, and fifth rows.

The estimated light sensitivity VFMs, the corresponding RMSE, standard deviation and average 68.2% HWCI for the simulated glaucoma observer, obtained from the qVFM-PDM methods are shown in Figure 5, along with the results from the qYN method. The corresponding results from the qVFM-DSM are shown in Supplementary Figure A2.

Compared with the simulated normal observer, the average light sensitivity deficit across all glaucoma damaged locations is 6.28 dB for the simulated glaucoma observer. Across the damaged locations, the average estimated deficits are 5.99 ± 0.77 dB and 6.13 ± 0.51 dB after 300 and 1,200 trials with the qVFM-DSM method, 6.32 ± 1.24 dB and 6.28 ± 0.59 dB after 300 and 1,200 trials with the qVFM-PDM method, and 6.16 ± 0.73 dB after 1,200 trials with the qYN method.

**FIGURE 6:**
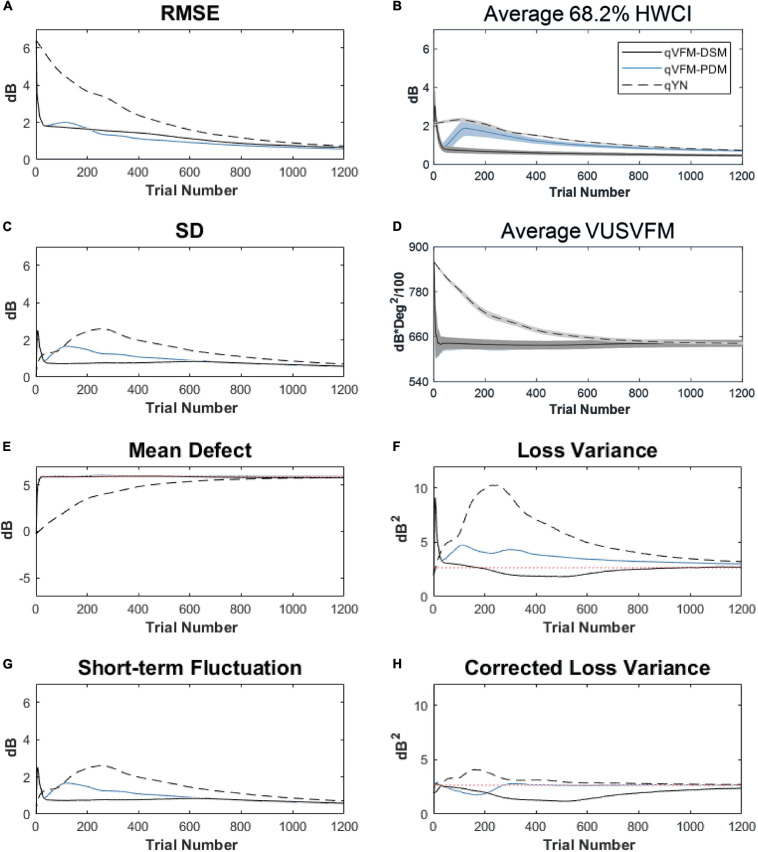
Performance I of the qVFM-DSM, qVFM-PDM and qYN methods in estimating VFM of the simulated glaucoma observer across 200 runs. **(A)** Average root mean squared error, **(B)** Average 68.2% HWCI of the estimated VFM, **(C)** Average standard deviation, **(D)** Average volume under the surface of the VFM (VUSVFM), **(E)** Mean defect, **(F)** Short-term fluctuation, **(G)** Loss variance, and **(H)** Corrected loss variance. Results from the qVFM-DSM and qVFM-PDM methods are shown in solid black and blue lines, and results from the qYN method are shown in dashed lines. The true values of the global indices are shown in red dotted lines. For panels **(B,D)**, shaded regions represent ± 1 SD of the corresponding value.

For the trial-by-trial performance, the RMSE, the average 68.2% HWCI, SD, the average VUSVFM, the mean defect, the loss variance, the short-term fluctuation, and the corrected loss variance of the estimated light sensitivity VFM from the three methods are shown in Figure 6, with numerical results listed in Table 3.

**TABLE 3:** RMSE, SD, average 68.2% HWCI, mean defect, loss variance and corrected loss variance of the estimated VFM from the qVFM-DSM, qVFM-PDM and qYN methods in the beginning (0 trial), after 300 and 1,200 trials are listed for the simulated glaucoma observer.

Glaucoma	Trial number	0	300	1,200	True value	1 dB/dB^2^ threshold
RMSE (dB)	qVFM-DSM	6.40	1.52	0.65		689 ± 35
	qVFM-PDM	6.40	1.30	0.58		522 ± 21
	qYN	6.40	3.13	0.73		909 ± 26
SD (dB)	qVFM-DSM	0	0.76	0.58		
	qVFM-PDM	0	1.23	0.57		
	qYN	0	2.45	0.71		
HWCI (dB)	qVFM-DSM	2.12	0.63	0.46		
	qVFM-PDM	2.12	1.44	0.71		
	qYN	2.12	1.68	0.74		
Mean defect (dB)	qVFM-DSM	*−*0.26	5.92	5.79	5.91	
	qVFM-PDM	*−*0.26	5.99	5.91	5.91	
	qYN	*−*0.26	4.22	5.79	5.91	
Loss variance (dB^2^)	qVFM-DSM	1.95	2.01	2.73	2.67	
	qVFM-PDM	1.95	4.43	3.02	2.67	
	qYN	1.95	9.23	3.22	2.67	
Corrected loss variance (dB^2^)	qVFM-DSM	1.95	1.43	2.38	2.67	677 ± 46
	qVFM-PDM	1.95	2.79	2.69	2.67	163 ± 15
	qYN	1.95	3.17	2.72	2.67	229 ± 26

*The corresponding true values are listed in the sixth column. Trial numbers needed to achieve 1 dB RMSE and 1 dB^2^ within the corrected loss variance are listed with their SD in the last column.*

For this simulated glaucoma observer, the qVFM-PDM method exhibited better performance than the qVFM-DSM method after 300 trials. The SD and short-term fluctuation of the estimated VFM obtained from the qVFM-PDM method are smaller, with the corrected loss variance approaching to the true value faster, compared with the qVFM-DSM method. Both qVFM methods demonstrated better efficiency than the qYN method. The trial numbers needed to achieve 1 dB RMSE and 1 dB^2^ within the corrected loss variance are shown in Table 3 for the three methods.

**FIGURE 7:**
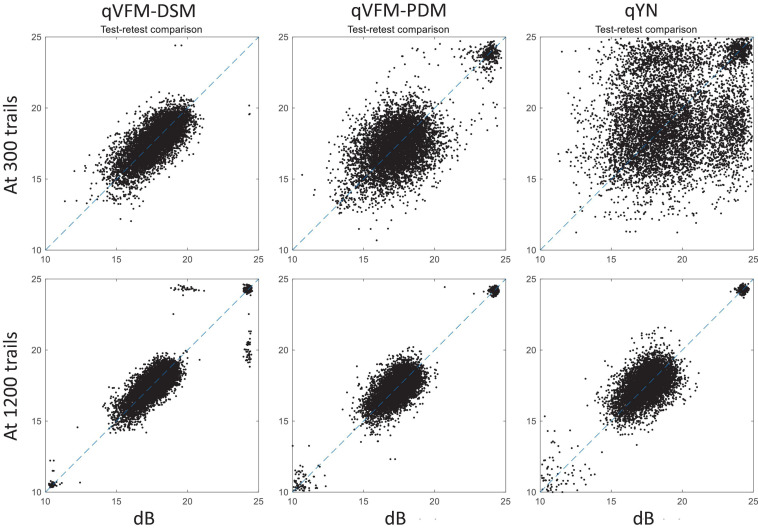
Performance II of the three methods in estimating VFM of the simulated glaucoma observer. Test-retest comparison of the estimated light sensitivities from repeated 200 runs at 300 and 1,200 trials.

For the simulated glaucoma observer, test–retest reliabilities of the three methods are assessed through analysis of VFM estimates at 300 and 1,200 trials across 200 runs (Figure 7). The average test–retest correlations for the paired VFM estimates at 300 trials are 0.725 (SD = 0.006) for the qVFM-DSM, 0.694 (SD = 0.006) for the qVFM-PDM and 0.274 (SD = 0.01) for the qYN methods, respectively. The average correlations at 1,200 trials are 0.917 (SD = 0.002) for the qVFM-DSM, 0.917 (SD = 0.002) for the qVFM-PDM and 0.875 (SD = 0.003) for the qYN methods, respectively.

### Simulated AMD Observer

**FIGURE 8:**
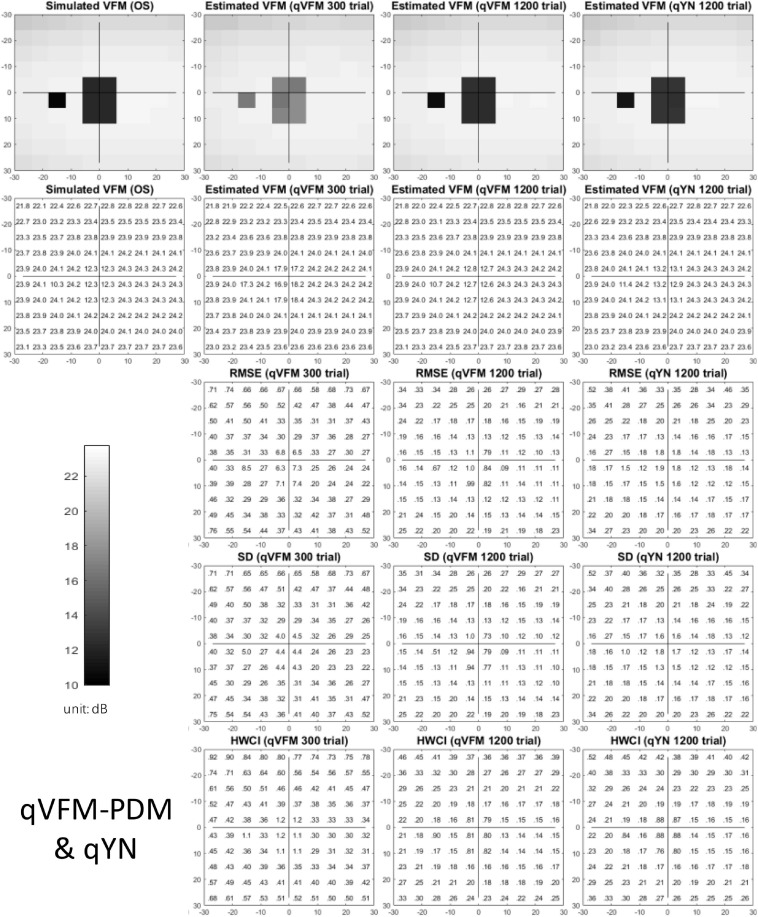
Simulation results I of the AMD observer across 200 runs. The true VFM of the simulated observer (monocular) is presented in the first column of the first row with achromatic colormaps and second row with numerical values. The estimated VFMs obtained with the qVFM-PDM method after 300 trials and 1,200 trials, qYN method after 1,200 trials are presented in the first and second rows, respectively. The corresponding RMSE, SD, and 68.2% HWCI of the estimates are shown in the third, fourth, and fifth rows.

The estimated light sensitivity VFMs, the corresponding RMSE, standard deviation and average 68.2% HWCI for the simulated AMD observer, obtained from the qVFM-PDM methods are shown in Figure 8, along with the results from the qYN method. The corresponding results from the qVFM-DSM are in shown in Supplementary Figure A3.

Compared with the simulated normal observer, the average light sensitivity deficit across all AMD damaged VF locations is 12.0 dB for the simulated AMD observer. Across all the damaged locations, the average estimated deficits are 7.60 ± 5.69 dB and 11.8 ± 1.64 dB after 300 and 1,200 trials with the qVFM-DSM method, 6.52 ± 4.38 dB and 11.6 ± 0.87 dB after 300 and 1,200 trials with the qVFM-PDM method, and 11.2 ± 1.64 dB after 1,200 trials with qYN method.

**FIGURE 9:**
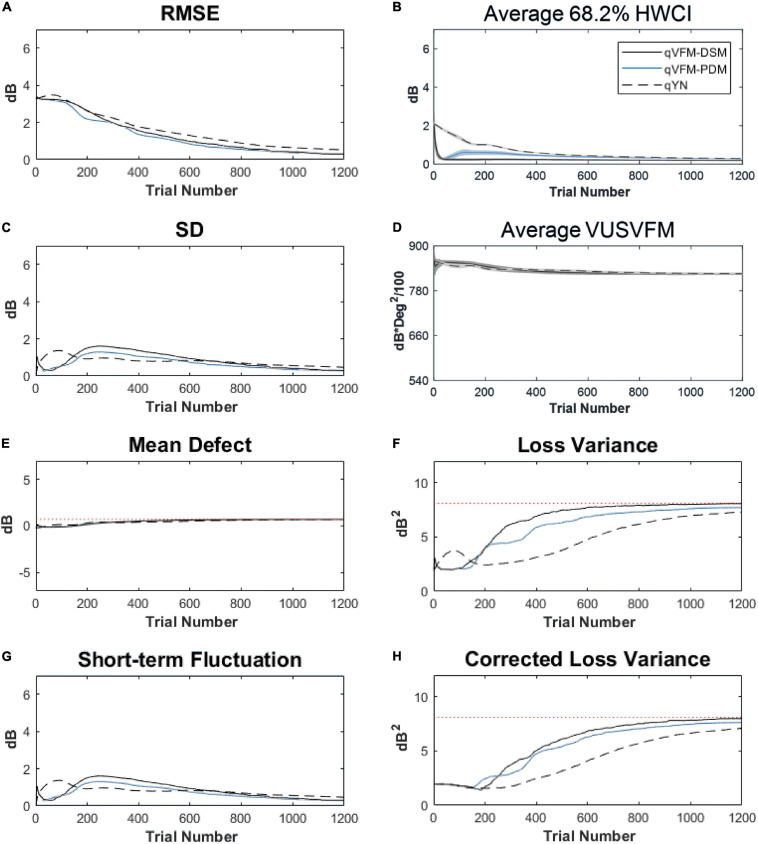
Performance I of the three methods in estimating VFM of the simulated AMD observer across 200 runs. **(A)** Average root mean squared error, **(B)** Average 68.2% HWCI of the estimated VFM, **(C)** Average standard deviation, **(D)** Average volume under the surface of the VFM (VUSVFM), **(E)** Mean defect, **(F)** Short-term fluctuation, **(G)** Loss variance, and **(H)** Corrected loss variance. Results from the qVFM-DSM and qVFM-PDM methods are shown in solid black and blue lines, and results from the qYN method are shown in dashed lines. The true values of the global indices are shown in red dotted lines. For panels **(B,D)**, shaded regions represent ± 1 SD of the corresponding value.

For the trial-by-trial performance, the RMSE, the average 68.2% HWCI, SD, the average VUSVFM, the mean defect, the loss variance, the short-term fluctuation, and the corrected loss variance of the estimated VFM from the three methods are shown in Figure 9, with numerical values listed in Table 4.

**TABLE 4:** RMSE, SD, average 68.2% HWCI, mean defect, loss variance and corrected loss variance of the estimated VFM from the qVFM-DSM, qVFM-PDM and qYN methods in the beginning (0 trial), after 300 and 1,200 trials are listed for the simulated AMD observer.

AMD	Trial number	0	300	1,200	True value	1 dB/dB^2^ threshold
RMSE (dB)	qVFM-DSM	3.26	1.98	0.30		591 ± 62
	qVFM-PDM	3.26	1.95	0.30		551 ± 18
	qYN	3.26	2.23	0.52		749 ± 28
SD (dB)	qVFM-DSM	0	1.55	0.30		
	qVFM-PDM	0	1.24	0.28		
	qYN	0	0.95	0.47		
HWCI (dB)	qVFM-DSM	2.12	0.24	0.19		
	qVFM-PDM	2.12	0.53	0.27		
	qYN	2.12	0.76	0.29		
Mean defect (dB)	qVFM-DSM	*−*0.26	0.40	0.71	0.72	
	qVFM-PDM	*−*0.26	0.40	0.71	0.72	
	qYN	*−*0.26	0.37	0.69	0.72	
Loss variance (dB^2^)	qVFM-DSM	1.95	6.18	8.08	8.11	
	qVFM-PDM	1.95	4.50	7.71	8.11	
	qYN	1.95	2.65	7.32	8.11	
Corrected loss variance (dB^2^)	qVFM-DSM	1.95	3.74	7.99	8.11	665 ± 66
	qVFM-PDM	1.95	2.93	7.63	8.11	822 ± 65
	qYN	1.95	1.74	7.10	8.11	1,198 ± 24

*The corresponding true values are listed in the sixth column. Trial numbers needed to achieve 1 dB RMSE and 1 dB^2^ within the corrected loss variance are listed with their SD in the last column.*

For the simulated AMD observer, the results showed that the qVFM-DSM and qVFM-PDM methods have similar performance. Both qVFM-DSM and qVFM-PDM methods demonstrated better efficiency than the qYN method. The trial numbers needed to achieve 1 dB RMSE and 1 dB^2^ within the corrected loss variance are shown in Table 4 for the three methods.

**FIGURE 10:**
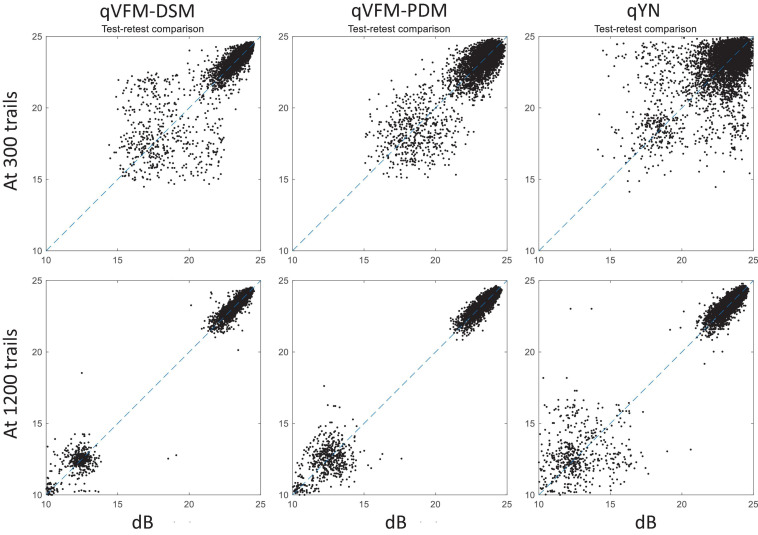
Performance II of the qVFM-DSM, qVFM-PDM and qYN methods in estimating VFM of the simulated AMD observer. Test-retest comparison of the estimated light sensitivities from repeated 200 runs at 300 and 1,200 trials.

For the simulated AMD observer, test–retest reliabilities of the three methods are assessed through analysis of VFM estimates at 300 and 1,200 trials across 200 runs (Figure 10). The average test–retest correlations for the paired VFM estimates at 300 trials are 0.868 (SD = 0.003) for the qVFM-DSM, 0.876 (SD = 0.003) for the qVFM-PDM and 0.585 (SD = 0.008) for the qYN method, respectively. The average correlations at 1,200 trials are 0.994 (SD = 0.0001) for the qVFM-DSM, 0.991 (SD = 0.0002) for the qVFM-PDM and 0.974 (SD = 0.001) for the qYN methods, respectively.

### Simulated Cataract Observer

**FIGURE 11:**
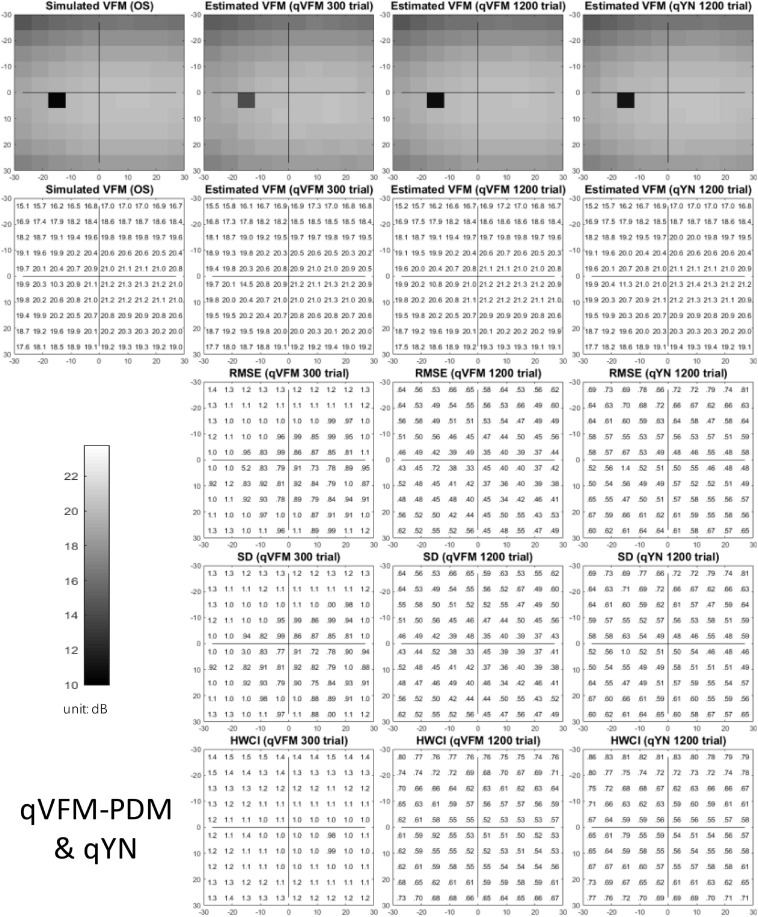
Simulation results I of the cataract observer across 200 runs. The true VFM of the simulated observer (monocular) is presented in the first column of the first row with achromatic colormaps and second row with numerical values. The estimated VFMs obtained with the qVFM-PDM method after 300 trials and 1,200 trials, qYN method after 1,200 trials are presented in the first and second rows, respectively. The corresponding RMSE, SD, and 68.2% HWCI of the estimates are in the third, fourth, and fifth rows.

The estimated light sensitivity VFMs, the corresponding RMSE, standard deviation and average 68.2% HWCI for the simulated cataract observer, obtained from the qVFM-PDM methods are shown in Figure 11, along with the results from the qYN method. The corresponding results from the qVFM-DSM are shown in Supplementary Figure 4.

**FIGURE 12:**
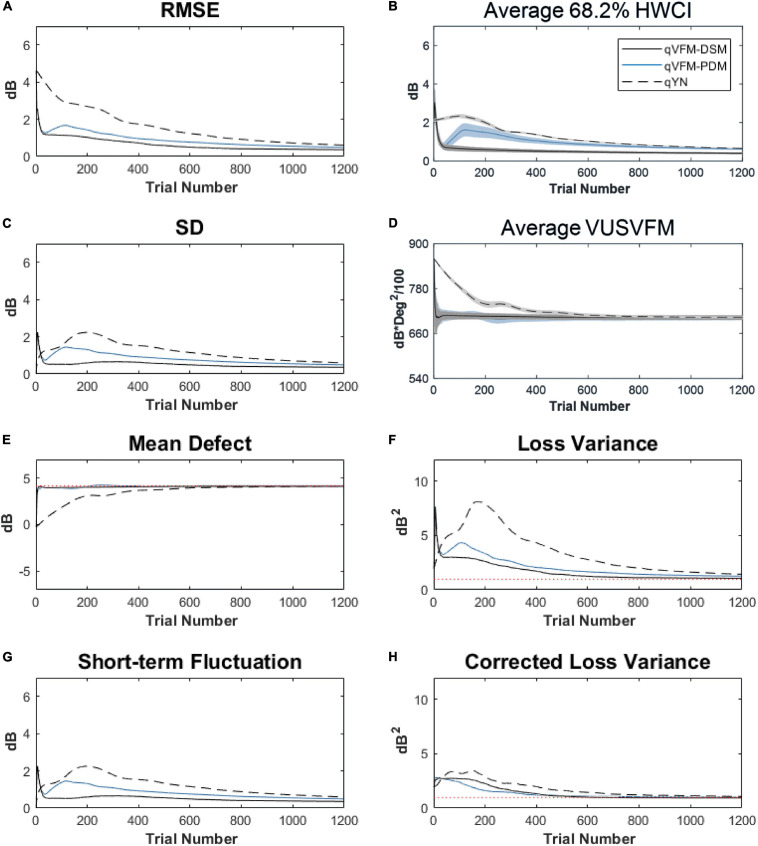
Performance I of the three methods in estimating VFM of the simulated cataract observer across 200 runs. **(A)** Average root mean squared error, **(B)** Average 68.2% HWCI of the estimated VFM, **(C)** Average standard deviation, **(D)** Average volume under the surface of the VFM (VUSVFM), **(E)** Mean defect, **(F)** Short-term fluctuation, **(G)** Loss variance, and **(H)** Corrected loss variance. Results from the qVFM-DSM and qVFM-PDM methods are shown in solid black and blue lines, and results from the qYN method are shown in dashed lines. The true values of the global indices are shown in red dotted lines. For panels **(B,D)**, shaded regions represent ± 1 SD of the corresponding value.

For the trial-by-trial performance, the RMSE, the average 68.2% HWCI, SD, the average VUSVFM, the mean defect, the loss variance, the short-term fluctuation, and the corrected loss variance of the estimated VFM from the three methods are shown in Figure 12, with some numerical values listed in Table 5.

**TABLE 5:** RMSE, SD, average 68.2% HWCI, mean defect, loss variance and corrected loss variance of the estimated VFM from the qVFM-DSM, qVFM-PDM and qYN methods in the beginning (0 trial), after 300 and 1,200 trials are listed for the simulated cataract observer.

Cataract	Trial number	0	300	1,200	True value	1 dB/dB^2^ threshold
RMSE (dB)	qVFM-DSM	4.63	0.85	0.37		212 ± 22
	qVFM-PDM	4.63	1.17	0.49		372 ± 27
	qYN	4.63	2.15	0.61		733 ± 29
SD (dB)	qVFM-DSM	0	0.65	0.36		
	qVFM-PDM	0	1.08	0.49		
	qYN	0	1.77	0.60		
HWCI (dB)	qVFM-DSM	2.12	0.55	0.40		
	qVFM-PDM	2.12	1.23	0.62		
	qYN	2.12	1.51	0.66		
Mean defect (dB)	qVFM-DSM	*−*0.26	4.04	4.12	4.16	
	qVFM-PDM	*−*0.26	4.20	4.18	4.16	
	qYN	*−*0.26	3.26	4.10	4.16	
Loss variance (dB^2^)	qVFM-DSM	1.95	2.04	1.07	0.98	
	qVFM-PDM	1.95	2.63	1.26	0.98	
	qYN	1.95	5.44	1.43	0.98	
Corrected loss variance (dB^2^)	qVFM-DSM	0	1.61	0.94	0.98	230 ± 24
	qVFM-PDM	0	1.45	1.01	0.98	142 ± 14
	qYN	0	2.27	1.07	0.98	401 ± 31

*The corresponding true values are listed in the sixth column. Trial numbers needed to achieve 1 dB RMSE and 1 dB^2^ within the corrected loss variance are listed with their SD in the last column.*

For the simulated cataract observer, the results showed that the qVFM-DSM and qVFM-PDM methods had similar performance. The RMSE of the estimated VFM from qVFM-DSM method is lower than that from the qVFM-PDM method, while the SD and short-term fluctuation from the qVFM-PDM are smaller than those from the qVFM-DSM method. Both qVFM methods demonstrated better efficiency than the qYN method. The trial numbers needed to achieve 1 dB RMSE and 1 dB^2^ within the corrected loss variance are shown in Table 5 for the three methods.

**FIGURE 13:**
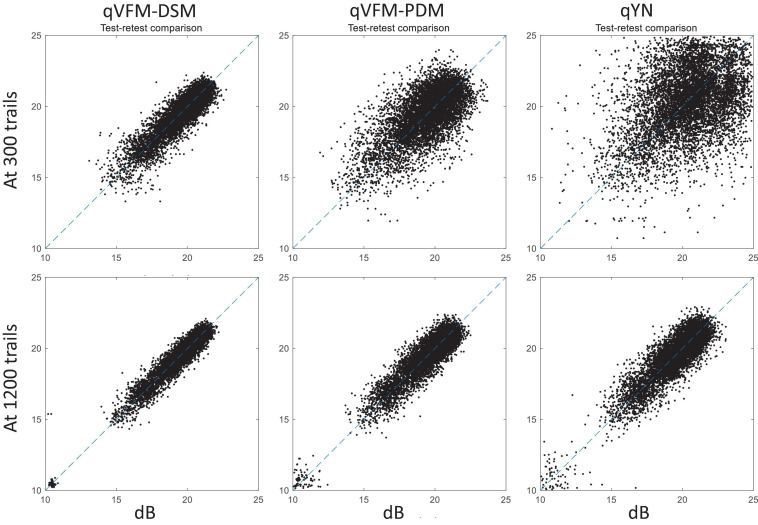
Performance II of the qVFM-DSM, qVFM-PDM and qYN methods in estimating VFM of the simulated cataract observer. Test-retest comparison of the estimated light sensitivities from repeated 200 runs at 300 and 1,200 trials.

For the simulated cataract observer, test–retest reliabilities of the three methods are assessed through analysis of VFM estimates at 300 and 1,200 trials across 200 runs (Figure 13). The average test–retest correlations for the paired VFM estimates at 300 trials are 0.89 (SD = 0.002) for the qVFM-DSM, 0.667 (SD = 0.007) for the qVFM-PDM and 0.389 (SD = 0.01) for the qYN methods, respectively. The average correlations at 1,200 trials are 0.96 (SD = 0.001) for the qVFM-DSM, 0.92 (SD = 0.002) for the qVFM-PDM and 0.881 (SD = 0.003) for the qYN method, respectively.

### Simulated Normal Observer

**FIGURE 14:**
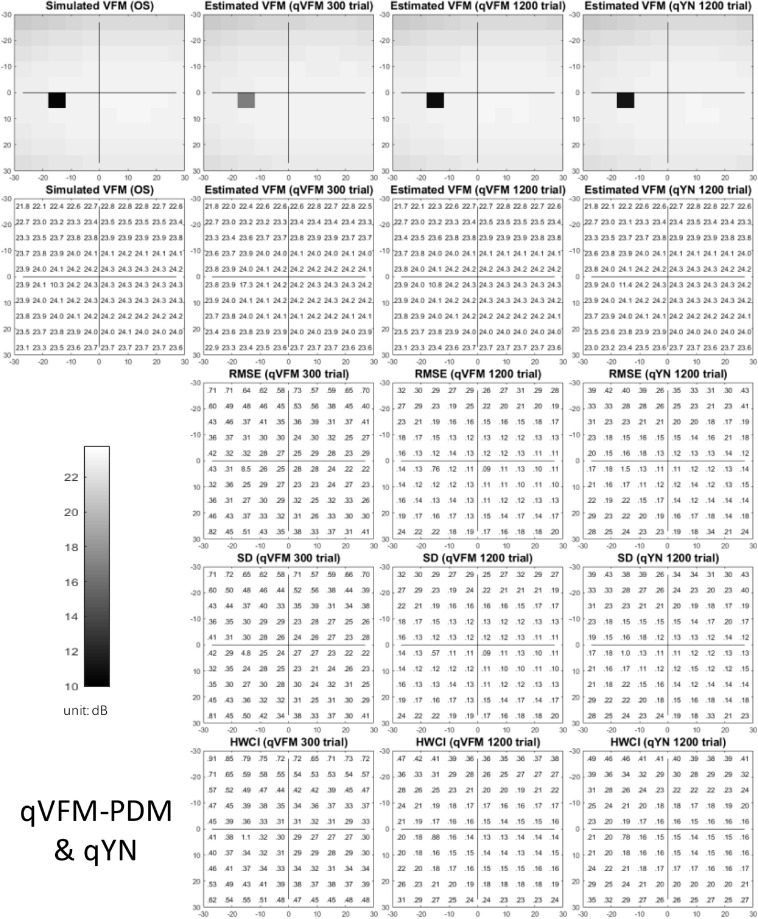
Performance I of the qVFM-DSM, qVFM-PDM and qYN methods in estimating VFM of the simulated normal observer across 200 runs. **(A)** Average root mean squared error, **(B)** Average 68.2% HWCI of the estimated VFM, **(C)** Average standard deviation, **(D)** Average volume under the surface of the VFM (VUSVFM), **(E)** Mean defect, **(F)** Short-term fluctuation, **(G)** Loss variance, and **(H)** Corrected loss variance. Results from the qVFM-DSM and qVFM-PDM methods are shown in solid black and blue lines, and results from the qYN method are shown in dashed lines. The true values of the global indices are shown in red dotted lines. For panels **(B,D)**, shaded regions represent ± 1 SD of the corresponding value.

The estimated light sensitivity VFMs, the corresponding RMSE, standard deviation and average 68.2% HWCI for the simulated normal observer, obtained from the qVFM-PDM methods are shown in Figure 14, along with the results from the qYN method. The corresponding results from the qVFM-DSM are shown in Supplementary Figure 5.

The RMSE, the average 68.2% HWCI, SD, the average VUSVFM, the mean defect, the loss variance, the short-term fluctuation, and the corrected loss variance of the estimated light sensitivity VFM from the three methods are shown in Supplementary Figure 6, with some numerical values listed in Table 6.

**TABLE 6:** RMSE, SD, average 68.2% HWCI, mean defect, loss variance and corrected loss variance of the estimated VFM from the qVFM-DSM, qVFM-PDM and qYN methods in the beginning (0 trial), after 300 and 1,200 trials are listed for the simulated normal observer.

Normal	Trial number	0	300	1,200	True value	1 dB/dB^2^ threshold
RMSE (dB)	qVFM-DSM	1.41	0.77	0.15		216 ± 28
	qVFM-PDM	1.41	0.94	0.19		267 ± 35
	qYN	1.41	1.16	0.27		353 ± 16
SD (dB)	qVFM-DSM	0	0.63	0.15		
	qVFM-PDM	0	0.62	0.18		
	qYN	0	0.75	0.24		
HWCI (dB)	qVFM-DSM	2.12	0.20	0.15		
	qVFM-PDM	2.12	0.45	0.22		
	qYN	2.12	0.70	0.24		
Mean defect (dB)	qVFM-DSM	*−*0.26	*−*0.05	0	0	
	qVFM-PDM	*−*0.26	0	0.01	0	
	qYN	*−*0.26	0.12	0.02	0	
Loss variance (dB^2^)	qVFM-DSM	1.95	0.60	0.02	0	
	qVFM-PDM	1.95	0.89	0.04	0	
	qYN	1.95	1.35	0.07	0	
Corrected loss variance (dB^2^)	qVFM-DSM	1.95	0.19	0	0	161 ± 19
	qVFM-PDM	1.95	0.50	0	0	164 ± 16
	qYN	1.95	0.79	0.01	0	248 ± 32

*The corresponding true values are listed in the sixth column. Trial numbers needed to achieve 1 dB RMSE and 1 dB^2^ within the corrected loss variance are listed with their SD in the last column.*

For the simulated normal observer, the performance of the qVFM-DSM and qVFM-PDM methods is similar. The RMSE and loss variance of both qVFM-DSM and qVFM-PDM methods drop quickly below those of the qYN method from the beginning, while those of the qYN method exhibit fluctuations. The trial numbers, needed to achieve 1 dB RMSE and 1 dB^2^ within the corrected loss variance, are shown in Table 6 for the three methods.

For the simulated normal observer, test–retest reliabilities of the three methods are assessed through analysis of VFM estimates at 300 and 1,200 trials across 200 runs (Supplementary Figure 7). The average test–retest correlations for the paired VFM estimates at 300 trials are 0.901 (SD = 0.002) for the qVFM-DSM, 0.784 (SD = 0.005) for the qVFM-PDM and 0.507 (SD = 0.009) for the qYN methods, respectively. The average correlations at 1,200 trials are 0.99 (SD = 0.001) for the qVFM-DSM, 0.983 (SD = 0.0004) for the qVFM-PDM and 0.969 (SD = 0.001) for the qYN methods, respectively.

## Discussion

In this study, we tested the performance of the qVFM method with two different switch modules, the DSM and PDM, along with the qYN method, in estimating the light sensitivity VFM of simulated observers with peripheral scotoma, glaucoma, AMD, cataract and normal vision. The results show that, whereas all three methods could provide accurate and precise assessment of VFM deficits, the qVFM method with both switch modules were more efficient than the qYN method. The qVFM-DSM and qVFM-PDM methods exhibited comparable performance in most cases, but the qVFM-PDM method was better at detecting vision loss in the simulated glaucoma. The results demonstrated the potential of the qVFM method in clinical applications.

### Comparison With Staircase-Based and SITA Algorithms

Most of the existing algorithms for static automated perimetry (SAP) are based on the staircase strategy (Weijland et al., 2004). In these algorithms, stimulus intensities are varied according to an up-and-down bracketing procedure in each location. The threshold values are estimated directly or scaled from the last seen stimulus intensity or the average of the last seen and unseen stimulus intensities in each location. In addition, the test procedures usually start from measuring thresholds at four primary points, one in each quadrant of the visual field, and followed by measurements of thresholds in the rest of the visual field with initial values based on the results at the primary points.

The conventional method with the Humphrey Field Analyzer is “Full Threshold,” which is currently regarded as the standard technique in SAP. With initial stimulus intensity levels determined from a normative data set, the stimulus intensity at each test location is varied in steps of 4 dB until the first response reversal occurs and then subsequently varied in steps of 2 dB, referred as the 4-2 dB staircase procedure. The stimulus intensity of the last-seen presentation is taken as the final threshold estimate, after a second response reversal has occurred at a given location (Artes et al., 2002). The other conventional method implemented in OCTOPUS perimeters uses a 4-2-1 dB staircase procedure, which further reduces the step size to 1 dB after two reversals. The mean value of the dimmest stimulus seen and the brightest stimulus not seen is defined as the threshold (Morales et al., 2000).

**FIGURE 15:**
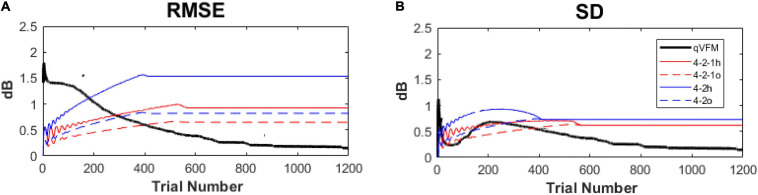
Performance comparison between the qVFM method and staircase procedures in simulation tests across 1,000 runs. **(A)** Average root mean squared error, **(B)** Average standard deviation. Results from the qVFM are shown in black solid lines. Results from the staircase 4-2-1 algorithm of h and o methods are shown in red solid and dashed lines. Results from the staircase 4-2 algorithm of h and o methods are shown in blue solid and dashed lines. The h method uses the last seen stimulus intensity as the estimated threshold, the o method uses the average of the last seen and unseen stimulus intensities as the estimated threshold.

In our previous study (Xu et al., 2019a), we compared the performance of the qVFM method and the staircase procedures, including both the 4-2 and 4-2-1 algorithms. The simulation results showed that, even with the initial stimulus intensity at each location matched with the true threshold of the simulated observer in staircase procedures, the staircase algorithms used in conventional SAP procedures still led to obvious biases and variabilities (Figure 15).

Another test algorithm, SITA (Swedish Interactive Thresholding Algorithm), can produce the same quality of test results as the Full Threshold strategy with considerable reduction of test time. However, it can only be used with the Goldmann III size stimulus of the Humphrey perimeter, and was only released for glaucomatous patients because it’s *a priori* threshold distribution was based on glaucoma (Bengtsson and Heijl, 1998; Artes et al., 2002). In our previous study (Xu et al., 2019a), we compared the SITA family, including SITA standard, SITA Fast and SITA Faster, with the qVFM method. Although both the SITA and the qVFM methods are based on the Bayesian adaptive testing framework, the qVFM is quite different from the SITA:

(1) Stimulus selection in the SITA family follows the conventional up-down staircase algorithm in each location with the stepsize no smaller than 2 dB, and the test follows the “grown pattern” procedure across the visual field (Bengtsson et al., 1997). In the qVFM method, the stepsize in the stimulus space can be as small as 0.12 dB. Both the global and local modules use the one-step-ahead search strategy across the entire visual field to determine the optimal stimulus in the next trial that would lead to the minimum expected entropy, equivalent to maximizing information gain on the next trial. More precise stimulus intensity and location selection in the qVFM method potentially leads to more accurate threshold estimation.
(2) The frequency-of-seeing curves (FOS-curves, a YN psychometric function) in the SITA is not adjusted with the observer’s decision criterion (Bengtsson et al., 1997). Previous studies showed that the conventional YN threshold estimates exhibit approximately 25–50% more variability (i.e., standard deviation) than the criterion-free forced-choice threshold estimates (McKee et al., 1985; King-Smith et al., 1994). In this study, we implemented the qVFM method based on the qYN procedure, which combines elements of Signal Detection Theory (SDT) and Bayesian adaptive inference to concurrently estimate thresholds associated with a d’ level (rather than a percent yes level) and decision criterion (Lesmes et al., 2015). Our study showed this method can deliver criterion-free thresholds on the light detection YN perimetric task with significant performance improvements.
(3) The prior distribution in the SITA is only optimized for normal and glaucoma observers, not for patients with other eye diseases (Bengtsson and Heijl, 1998). In the qVFM, the global module performs an individualized test that takes into account of the population properties in its prior but continues to optimize the test for each individual, including patients with abnormal VFMs. Because the method is completely systematic and not oriented toward any particular pathological pattern, the qVFM is not limited to a specific eye disease.
(4) Because locations with defective visual sensitivity tend to appear in clusters in glaucomatous visual fields, the prior threshold distribution in each test location in the SITA is calculated with inter-location correlations (Bengtsson et al., 1997). Such correlations may not be present in other eye diseases. In the qVFM, the local module is used to estimate visual threshold in each visual field location independently, making it possible to detect steep visual sensitivity changes in the visual field, such as scotoma resulting from optic neuropathies or visual field islands in retinitis pigmentosa.
(5) Since the age corrected normal and glaucomatous priors need enormous data collection to generate, the development of the SITA has so far been focused on light sensitivity maps with Goldmann III size stimulus for the 30-2, 24-2, 10-2 test patterns in the Humphrey Field Analyzer (Bengtsson et al., 1997; Phu et al., 2017). Whereas the test area in the SITA is limited to the central 30 degrees of the visual field with less than 76 test locations, the qVFM can map larger areas of the visual field with different type of stimulus and a flexible number of test locations, without restricts from prior knowledge as SITA.

### Alternative Methods for the Switch Module

In previous studies (Xu et al., 2018, 2019a,b, 2020), we generated the prior distributions in the local module by sampling the posterior from the global module using the qVFM-DSM method. The method was used to effectively transmit information obtained from the global module to the local module. However, it might generate priors that are too strong and hinder the detection of local VF deficits by the local module. In this study, we developed a new switch model, the qVFM-PDM mothed. It marginalizes the posterior distributions of the six parameters from the global module, and uses the average 68.2% HWCI of the estimated sensitivities and decision criterion across all VF locations to assign priors in the local module. By averaging estimated variabilities across all the VF locations to generate the prior distribution for each location in the local module, the qVFM-PDM is more robust and better enables the local module to detect local VF deficits. Our results showed that, although both the qVFM-DSM and qVFM-PDM methods performed well in estimating the VFM of the simulated observes, the qVFM-PDM method exhibited better performance in detecting VF loss in the simulated glaucoma in this study.

In these implementations, the rate of information gain in the global module was used to compute the switching point. Alternative methods could be used to determine the switching point, such as the relative amount of information gain from the global and local modules, a criterion based on the HWCI of the estimated VFM, or the convergence of parameters in the global module. Constrained by the amount of computation required to maintain and update the various probability distributions in both the global and local modules, we only performed a one-way switch from the global module to the local module in the current study. With additional computing power or better algorithms, we might be able to switch between the two modules multiple times if necessary.

### Paths for Potential Extension

In this study, we took a very conservative approach in setting the prior for all the simulated observers. The prior was in fact mis-informative for the simulated observers with peripheral scotoma, glaucoma, AMD, and cataract. As a result, the qVFM method exhibited worse performance in the beginning of the estimating process. A hierarchical Bayes extension of qVFM could be developed to provide a judicious way to derive informative priors for different patient populations (Kim et al., 2014; Gu et al., 2016): An incoming patient is assigned to some possible disease categories, each with its own prior distributions. The hierarchical qVFM would update both category probabilities and the distribution of the VFM parameters during the test, and update the prior of each disease category after testing each patient. Alternatively, or jointly within the hierarchical framework, the prior in the qVFM could be informed by knowledge obtained from each patient’s pervious diagnoses.

The qVFM method provides a general framework for mapping many other visual functions, such as visual acuity, CSF, color, stereo vision, reading speed, motion sensitivity, temporary sensitivity, and crowding. Once developed, measurements of the multiple VFMs would allow us to analyze and model the relationships among multiple visual functions as well as performance in everyday visual tasks, and identify the core metrics of functional vision deficits in patients with eye disease.

The broad adoption of the qVFM method would also require development of less expensive and integrated devices. Given the current development of cost-effective eye trackers and rapid improvement of consumer technology, we are optimistic that this could be accomplished in the near future.

## Conclusion

In this study, we showed that the qVFM method can be used to characterize residual vision of simulated ophthalmic patients. It sets the stage for further investigation with real patients. We anticipate that the qVFM method, with additional tests on real patients, can be potentially translated into clinical practice in the future.

## Data Availability Statement

The original contributions presented in the study are included in the article/Supplementary Material, further inquiries can be directed to the corresponding author.

## Author Contributions

Z-LL, PX, LL, and DY designed the qVFM algorithms. PX performed simulations and analyzed the data. PX and Z-LL wrote the manuscript with input from all authors. Z-LL and DY supervised the project. All authors contributed to the article and approved the submitted version.

## Conflict of Interest

PX, LL, DY, and Z-LL own intellectual property rights on the qVFM technology. LL and Z-LL have equity interest in Adaptive Sensory Technology, Inc. LL holds employment at Adaptive Sensory Technology, Inc. PX holds employment at Shanghai Technology Development Co., Ltd.
