# Supplementary material for: Evaluating the Performance of qVFM in Mapping the Visual Field of Simulated Observers With Eye Diseases
Source: Front Neurosci. 2021 Jun 21;15:596616. doi: 10.3389/fnins.2021.596616 (PMC8255634; doi:10.3389/fnins.2021.596616)
Supplement: Supplementary file 1 [file Data_Sheet_1.docx]

**Appendix A: Simulation results from the qVFM-DSM method**

The estimated light sensitivity VFMs, the corresponding RMSE, standard deviation and average 68.2% HWCI, obtained from the qVFM-DSM method are shown in Figures 1 for the simulated scotoma observer, Figures 2 for the simulated Glaucoma observer, Figures 3 for the simulated AMD observer, Figures 4 for the simulated Cataract observer, Figures 5 for the simulated normal observer, along with the corresponding results from the qYN method. The performance results of the qVFM-DSM, qVFM-PDM and qYN methods for the simulated normal observer are shown in Figure 6. Test-retest correlations of the three methods for the simulated normal observer are shown in Figure 7.

**Figure 1:** Simulation results II of the scotoma observer across 200 runs. The true VFM of the simulated observer (monocular) is presented in the first column of the first row with achromatic colormaps and second row with numerical values. The estimated VFMs obtained with the qVFM-DSM method after 300 trials and 1200 trials, qYN method after 1200 trials are presented in the first and second rows, respectively. The corresponding RMSE, SD, and 68.2% HWCI of the estimates are in the third, fourth, and fifth rows.

**Figure 2:** Simulation results II of the glaucoma observer across 200 runs. The true VFM of the simulated observer (monocular) is presented in the first column of the first row with achromatic colormaps and second row with numerical values. The estimated VFMs obtained with the qVFM-DSM method after 300 trials and 1200 trials, qYN method after 1200 trials are presented in the first and second rows, respectively. The corresponding RMSE, SD, and 68.2% HWCI of the estimates are in the third, fourth, and fifth rows.

**Figure 3:** Simulation results II of the AMD observer across 200 runs. The true VFM of the simulated observer (monocular) is presented in the first column of the first row with achromatic colormaps and second row with numerical values. The estimated VFMs obtained with the qVFM-DSM method after 300 trials and 1200 trials, qYN method after 1200 trials are presented in the first and second rows, respectively. The corresponding RMSE, SD, and 68.2% HWCI of the estimates are in the third, fourth, and fifth rows.

**Figure 4:** Simulation results II of the cataract observer across 200 runs. The true VFM of the simulated observer (monocular) is presented in the first column of the first row with achromatic colormaps and second row with numerical values. The estimated VFMs obtained with the qVFM-DSM method after 300 trials and 1200 trials, qYN method after 1200 trials are presented in the first and second rows, respectively. The corresponding RMSE, SD, and 68.2% HWCI of the estimates are in the third, fourth, and fifth rows.

**Figure 5:** Simulation results II of the normal observer across 200 runs. The true VFM of the simulated observer (monocular) is presented in the first column of the first row with achromatic colormaps and second row with numerical values. The estimated VFMs obtained with the qVFM-DSM method after 300 trials and 1200 trials, qYN method after 1200 trials are presented in the first and second rows, respectively. The corresponding RMSE, SD, and 68.2% HWCI of the estimates are in the third, fourth, and fifth rows.

 **Figure 6:** Performance I of the qVFM-DSM, qVFM-PDM and qYN methods in estimating VFM of the simulated normal observer across 200 runs. (a) Average root mean squared error, (b) Average 68.2% HWCI of the estimated VFM, (c) Average standard deviation, (d) Average volume under the surface of the VFM (VUSVFM), (e) Mean defect, (f) Short-term fluctuation, (g) Loss variance, and (h) Corrected loss variance. Results from the qVFM-DSM and qVFM-PDM methods are shown in solid black and blue lines, and results from the qYN method are shown in dashed lines. The true values of the global indices are shown in red dotted lines. For (b) and (d), shaded regions represent ±1 SD of the corresponding value.

**Figure 7:** Performance II of the qVFM-DSM, qVFM-PDM and qYN methods in estimating VFM of the simulated normal observer. Test-retest comparison of the estimated light sensitivities from repeated 200 runs at 300 and 1200 trials.

**Appendix B: The qVFM method**

The qVFM method consists of three major modules. In the global module, the shape of the VFM is modeled as a tilted elliptic paraboloid function (TEPF) with five parameters. The score of the VFM at each visual field location represents a measure of functional vision at that location. In mapping light sensitivity, the score at each location of the VFM represents perceptual sensitivity (1/threshold) at a fixed d’ level at that location. Together with a decision criterion (the sixth parameter) and a slope of the psychometric function (assumed to be fixed), the VFM can be used to predict light detection probability at every single visual field location. Using Bayesian update and optimal stimulus selection (Kontsevich and Tyler, 1999; Lesmes et al., 2015) , the global module updates the joint posterior distribution of the six parameters, that is, the shape of the VFM and the decision criterion, based on subject’s response in each trial.

The switch module evaluates the rate of information gain in the global module and determines when to switch to the local module. At the point of the switch, the module generates a priori distribution of the measure of functional vision at each visual field location based on the posterior from the global module. In this study on mapping light sensitivity, the prior is over both light sensitivity and decision criterion at each visual field location.

Using the prior generated by the switch module, the local module provides assessment of functional vision at each visual field location using another Bayesian adaptive procedure that determines the order and test stimulus based on the relative information gain across locations. In mapping light sensitivity, we used qYN (Lesmes et al., 2015) as the adaptive procedure to assess visual function at each visual field location and the expected information gain across all visual field locations and stimulus intensity levels to determine the optimal test stimulus in each trial.

*The Global Module*

In the global module, we first define a six-dimensional prior probability distribution, in which the first five dimensions correspond to the five parameters of the tilted elliptic paraboloid function (TEPF) and the sixth dimension represents the decision criterion in a YN task in light detection. The prior distribution, together with the slope of the psychometric function, completely specifies the response probabilities in the detection task across all the light intensity levels and visual field locations of all possible observers. We then define a two-dimensional stimulus space, representing both the spatial locations and intensities of the stimuli. The optimal stimulus (location and light intensity) for the first test is determined based on the prior distribution. Bayes rule is used to update the posterior distribution of the six parameters based on the observer’s response. A new trial starts using the posterior from the previous trial as the prior. The procedure repeats until the switch module decides to switch to the local module.

Modeling the likelihood function of the VFM with a tilted elliptic paraboloid function

In the global module, we model the VFM as a TEPF of spatial location with five parameters (Eq.1): (1) central gain (e.g., sensitivity at the fovea), *EPZ*; (2) root bandwidth (latus rectum) in the horizontal direction, *EPA*, which describes the square root of function’s full-width at half-maximum (in octaves) in the horizontal direction of the visual field; (3) root bandwidth in the vertical direction, *EPB*; (4) tilted level in the horizontal direction, *SLA*; and (5) tilted level in the vertical direction, *SLB.*

In mapping light sensitivity, the height of the TEPF, $\tau(x,y)$, defines the light sensitivity (1/threshold) at a fixed *d’*=1.0 level at visual field location *(x,y)*. The *d'* psychometric function at each visual field location *(x,y)*, that is, perceptual sensitivity for a given stimulus intensity *s,* is modeled as:

$d'(s,x,y)=\frac{{\beta(s*\tau(x,y))}^{\gamma}}{\sqrt{{(s*\tau(x,y))}^{2\gamma}+(\beta^{2}-1)}},$ (B1)

where *s* is the intensity of the stimulus, *γ* is the slope of the *d′* psychometric function, and *β* is the asymptote of the function (Lesmes et al., 2015; Lu & Dosher, 2008). Plotted on log axes, this function is approximately linear over low to medium stimulus intensities and saturates at high intensities. Following the previous study (Lesmes et al., 2015), we have fixed *γ* (=2.1) and *β* (=5.0) in the current implementation of the qVFM.

In a YN detection task at a visual field location *(x,y)*, the probability of reporting target presence (“yes”) is determined by both perceptual sensitivity and decision criterion. Based on signal detection theory(Gu & Green, 1994; Klein, 2001):

$P\left( s,x,y \right)=\int_{-\infty}^{+\infty} \phi\left( t-{(d}^{'}\left( s,x,y \right)-\lambda\left( x,y \right)) \right)\Phi(t)dt,$ (B2)

where 𝜙() is the probability density function of a standard normal distribution function, $\Phi()$ is the cumulative probability density function of a standard normal distribution function, *d'(s, x, y)* is the *d'* value associated with a stimulus with signal intensity *s* at visual field location *(x,y)*, and *λ(x,y)* is the decision criterion at visual field location *(x,y)*. In the global module, a single *λ* is used across all visual field locations. In the local module, *λ(x,y)* is independent at each visual field location.

In addition, we assume a fixed lapse rate *ε* for human observers (Klein, 2001; Wichmann & Hill, 2001; Lesmes, et al, 2015):

$P^{'}\left( s,x,y \right)=\frac{1}{2}\varepsilon+ \left( 1-\varepsilon\right)P \left( s,x,y \right),$ (B3)

where *P(s,x,y)* is the psychometric function without lapse . In the qVFM method, *ε* was set to 0.03 (Lesmes et al., 2010; Wichmann & Hill, 2001).

Eq. B3 is the likelihood function of the VFM that completely describes the probability of light detection across all visual field locations and light intensity levels.

Setting the prior and stimulus space

Before running the qVFM procedure, a probability density function, $p(\vec{\theta})$, where $\vec{\theta}=\left( EPZ, EPA, EPB, SLA, SLB, \lambda\right),$is defined over the parameter space of the TEPF and decision criterion. Before any data collection (trial *t* = 0), the initial priori distribution *p_o_*$(\vec{\theta}$) represents foreknowledge of model parameters. In addition, a stimulus space, that includes all possible stimulus locations *(x, y)* and stimulus intensities is defined. With the prior and the stimulus space, we can compute the probability of detecting any stimulus in the visual field for all possible observers based on Eq. B3.

Stimulus selection

In the qVFM, information is quantified by entropy, a measure of uncertainty associated with random variables. The method uses a one-step-ahead search strategy to determine the optimal stimulus in the next trial that would lead to the minimum expected entropy. It first computes (1) the observer’s response probability *P_t+1_(r|s)* in every possible stimulus condition in the next trial based on the current prior, (2) the expected posterior probability distributions for all possible stimuli, and (3) the expected entropy for each possible stimulus.

The entropy of the posterior is defined as:

$H_{t+1}\left( s,r \right)=-\sum_{\vec{\theta}} P_{t+1}\left( \vec{\theta}|s,r \right)*log \left( P_{t+1}(\vec{\theta}|s,r) \right) ,$ (B4)

where *r* represents observer’s response (correct or incorrect) to a test with signal intensity *s*. The expected entropy after a trial with the signal intensity of stimulus, *s,* is calculated as a weighted sum of posterior entropy:

${E[H}_{t+1}\left( s,r \right)]=\sum_{r} H_{t+1}\left( s,r \right){*P}_{t+1}\left( r | s \right).$ (B5)

The stimulus with the minimum expected entropy is chosen for the next trial:

$s_{t+1}=\arg\min_{s} E[H_{t+1}\left( s \right)]$ . (B6)

This is equivalent to maximizing the expected information gain, quantified as the entropy change between the prior and posterior (Kujala and Lukka, 2006; Lesmes et al., 2006).

Bayesian update

The priori distribution *p_t_(*$\vec{\theta}$*)* in the *t-*th trial is updated to the posterior distribution *p_t_(*$\vec{\theta}$*|s,r_t_)* with the observer’s response *r_t_* (correct or incorrect) to a test with a stimulus, *s,* by Bayes rule:

$P_{t}\left( \vec{\theta} | s,r_{t} \right)=\frac{P\left( r_{t} | \vec{\theta},s \right)P_{t}(\vec{\theta})}{P_{t}\left( r_{t} | s \right)}$ , (B7)

where *θ* represents the parameters of the VFM model, *p_t_(*$\vec{\theta}$*)* is the prior probability function of $\vec{\theta}$. The probability of a response *r_t_* in a given stimulus condition *s*, *p_t_(r_t_|s)*, is estimated by weighting the empirical response probability by the prior:

$P_{t}\left( r_{t} | s \right)=\sum_{\vec{\theta}} [{P\left( r_{t} | \vec{\theta},s \right)P}_{t}(\vec{\theta})]$, (B8)

where *p(r_t_|s,*$\vec{\theta}$*)* is the likelihood of observing response *r_t_* given $\vec{\theta}$ and stimulus s; The posterior *p_t_(*$\vec{\theta}$*|s,r_t_)* following the *t-*th trial serves as the prior *p_t+1_(*$\vec{\theta}$*)* in the next trial:

$P_{t+1}(\vec{\theta})=P_{t}(\vec{\theta}|s,r_{t})$. (B9)

The means of the marginal posterior distributions are used to estimate the parameters of the qVFM model after each trial.

*The Switch Module*

Since TEPF does not provide detailed local measures of the VFM, we designed a switch module to transfer from the global module to the local module for further assessment.

In the global module, the expected information gain, computed as the difference between the entropy of the prior and the expected posterior distribution for each potential stimulus is computed before each trial. The stimulus that would lead to the maximum amount of information gain is used in the next trial. Before each trial, the expected information gain is also used by the switch module to determine the switch point to the local module. Instead of using the maximum expected information gain, the switch module computes the total expected information gain (TEI) from the top 10% potential stimuli. In the beginning, the TEI is high in the global module. With increasing number of trials, the TEI is expected to gradually decrease as the method learns more about the parameters. As the learning saturates over trials, the trend of TEI begins to flatten and may even reverse – that is, the TEI in trial t+1 may be higher than that of its previous trials. In the current implementation, the switch module compares the TEI in trial t+1 with the average TEI of three previous trials, t-2, t-1, and t to determine whether to switch to the local module. The switch happens when the TEI in trial t+1 is higher than the average in trial t-2, t-1 and t.

Upon the switch, the switch module generates a priori distribution of functional vision in each visual field location based on the posterior of the parameters in the global module. Specifically, two different methods were implemented in this study for generating the priori distribution for each visual field location.

In the distribution sampling method (DSM; Xu et al., 2018; 2019), the switch module samples the posterior distribution of the TEPF model repeatedly to generate the prior distributions of the light sensitivity in each visual location, with 1600 samples per location. In the newly developed parameter delivering method (PDM), the switch module computes the expected values of the five parameters of the TEPF model from their posterior distributions, and sets the expected values of the light sensitivity prior at each visual field location based on the TEPF model. It also sets the expected value of the prior of the decision criterion in each visual field location using its expected value from the posterior distribution in the global module. It then uses the average 68.2% half width of the credible interval (HWCI) of the posterior distributions of the estimated sensitivities and decision criterions across all visual field locations to set the variability of the prior distributions in the local module. Specifically, a hyperbolic secant (sech) function^28^ is used to set up the prior distribution. For each parameter *θ_i_* (i = 1, 2), the mode of the marginal prior *p(θ_i_)* was defined by the expected value of the corresponding parameter, *θ_i,guess_*, from the posterior distributions of the global module, and the width was defined by the 68.2% credible interval of that parameter.

$P\left( \theta_{i} \right)=sech(\theta_{i,confidence}\times(\theta_{i}-\theta_{i, guess}))$, (B10)

where

$\mathrm{sech} \left( z \right)=\frac{2}{e^{z}{+e}^{-z}}$. (B11)

The joint prior is defined as the normalized product of the marginal priors, which is generated for each visual field location.

*The Local Module*

The setup of the local module is very similar to that of the global module except the following:

1. Independent parameters at each visual field location: Instead of using six parameters to model visual function across all visual field locations, independent parameters are used to model visual function in each visual field location. In mapping light sensitivity, $\tau\left( x,y \right)$is no longer described by Eq. 1, but is rather independent at each location.
2. Independent priors and posteriors: Each visual field location has its independent parameters and therefore independent priors and posteriors. The initial priors in the local module are generated by the switch module.
3. Computing the information gain: The information gain of each location is computed independently, while in the global module the information gains are computed simultaneously across all visual field locations based on the tilted elliptic paraboloid function and the psychometric function. Regardless of the dependency of the computations of information gains, optimal stimulus selection is always based on the total expected entropy across all the visual field locations in both local and global modules. In other words, to select the next test location and stimulus intensity, the expected entropy from all visual field locations is considered.

*Stopping rules*

In the current implementation, the qVFM procedure terminates after a fixed number of trials. Alternatively, the qVFM procedure can stop after it achieves a certain defined objective (e.g., after reaching a criterion level of precision for either the parameters in qVFM or the light sensitivity across all visual field locations).
